# Quantitative Detection of Disseminated Melanoma Cells by Trp-1 Transcript Analysis Reveals Stochastic Distribution of Pulmonary Metastases

**DOI:** 10.3390/jcm10225459

**Published:** 2021-11-22

**Authors:** Lenka Kyjacova, Rafael Saup, Melanie Rothley, Anja Schmaus, Tabea Wagner, Anja Boßerhoff, Boyan K. Garvalov, Wilko Thiele, Jonathan P. Sleeman

**Affiliations:** 1 Department of Microvascular Biology and Pathobiology, European Center for Angioscience (ECAS), Medical Faculty Mannheim, University of Heidelberg, D-68167 Mannheim, Germany; lenka.kyjacova@gmail.com (L.K.); Rafael.Saup@medma.uni-heidelberg.de (R.S.); melanie.rothley@kit.edu (M.R.); anja.schmaus@partner.kit.edu (A.S.); Tabea.Wagner@medma.uni-heidelberg.de (T.W.); Boyan.Garvalov@medma.uni-heidelberg.de (B.K.G.); Wilko.Thiele@medma.uni-heidelberg.de (W.T.); 2 Institute for Biological and Chemical Systems-Biological Information Processing (IBCS-BIP), Karlsruhe Institute of Technology (KIT)-Campus North, D-76344 Karlsruhe, Germany; 3 Institute of Biochemistry, Faculty of Medicine, Friedrich-Alexander University Erlangen-Nürnberg (FAU), D-91054 Erlangen, Germany; anja.bosserhoff@fau.de

**Keywords:** Trp-1, metastasis, melanoma, animal models, lung, mice

## Abstract

A better understanding of the process of melanoma metastasis is required to underpin the development of novel therapies that will improve patient outcomes. The use of appropriate animal models is indispensable for investigating the mechanisms of melanoma metastasis. However, reliable and practicable quantification of metastases in experimental mice remains a challenge, particularly if the metastatic burden is low. Here, we describe a qRT-PCR-based protocol that employs the melanocytic marker Trp-1 for the sensitive quantification of melanoma metastases in the murine lung. Using this protocol, we were able to detect the presence of as few as 100 disseminated melanoma cells in lung tissue. This allowed us to quantify metastatic burden in a spontaneous syngeneic B16-F10 metastasis model, even in the absence of visible metastases, as well as in the autochthonous Tg(*Grm1*)/*Cyld^−/−^* melanoma model. Importantly, we also observed an uneven distribution of disseminated melanoma cells amongst the five lobes of the murine lung, which varied considerably from animal to animal. Together, our findings demonstrate that the qRT-PCR-based detection of Trp-1 allows the quantification of low pulmonary metastatic burden in both transplantable and autochthonous murine melanoma models, and show that the analysis of lung metastasis in such models needs to take into account the stochastic distribution of metastatic lesions amongst the lung lobes.

## 1. Introduction

Malignant cutaneous melanoma is characterized by early dissemination and subsequent metastatic colonization of multiple organs [1]. One of the most frequent sites of metastasis is the lung, with around 18% of melanoma patients developing metastatic pulmonary foci during follow-up [2,3], which is associated with a 1-year survival rate of 57% [4].

Experimentally, the process of melanoma metastasis is usually investigated using rodent models. However, accurate quantification of organ metastases in these models remains a significant challenge. Traditionally, visible superficial metastases are enumerated post mortem. However, metastases need to be relatively large to be quantified, and metastatic foci growing within the organ, that are not visible from the surface, will not contribute to the quantification. To circumvent these problems, organs are often also examined histologically. However, this is a laborious process, particularly if metastases are rare and entire organs must be sectioned to provide a systematic assessment of metastatic burden [5]. Immune cell infiltrates or perivascular areas may also be misidentified histologically as micrometastases [5]. Moreover, an unequal distribution of metastatic foci within a particular organ may lead to misleading quantification results with such histological methods. For example, the mouse lung has five lobes; if pulmonary metastasis is evaluated in only a single lobe, or if metastasis is quantified through multiple methods that each use a different lobe, then a skewed or misleading quantification may ensue if metastatic foci are not equally distributed throughout all of the lung lobes.

Several imaging technologies facilitate the evaluation of metastasis formation in specific organs, either longitudinally within living animals, or ex vivo post mortem. Photonic imaging methods based on fluorescence or bioluminescence have been widely employed, and while they offer a number of important advantages, they are also subject to a number of major limitations. Photon attenuation as a function of depth and of the heterogeneous optical properties of tissues can result in loss of signal and non-linear results, and signal scattering by tissues limits spatial resolution [6]. In addition, the luciferase proteins that catalyze the bioluminescence reaction, as well as fluorescent reporter proteins, such as GFP, can trigger immune responses in immunocompetent animals, leading to the elimination of luciferase- or GFP-expressing cells, and the suppression of metastasis formation [7,8,9]. Moreover, oxygen and ATP are required for the generation of a bioluminescence signal, and as tumor tissues are often hypoxic, this can significantly attenuate the signal generated [10]. Other imaging approaches, such as magnetic resonance imaging (MRI), X-ray computed tomography (CT), or positron emission tomography (PET) can be used for imaging and quantification of metastases, but are limited in their sensitivity and require specialized and costly equipment.

Therefore, there is a need for methods that allow reliable detection and quantification of overt metastases, micrometastases and disseminated tumor cells. PCR-based approaches have the potential to fulfil these requirements, if marker genes are expressed, specifically in metastatic cells and not in the tissues in which metastases develop [11,12,13,14,15,16,17,18]. Tyrosine-related proteins 1 and 2 (Trp-1 and Trp-2) are involved in the synthesis of melanin (reviewed in [19,20]), which is characteristic for cells of melanocytic origin. Accordingly, Trp-1 and Trp-2 transcripts are absent from most non-melanocytic tissues [21], but are expressed in melanocytes, in melanomas and their metastases, and by many different melanoma cell lines [17,22,23], including the well-established and widely used B16 cell line [22,24]. Therefore, Trp-1 and Trp-2 represent potentially useful markers for the detection of disseminated melanoma cells, over and above any endogenous expression from melanocytes in the tissue concerned [17].

Here, we describe the development of a protocol for the detection of melanoma cells in the lung of experimental mice, based on quantification of tyrosine-related protein 1 (Trp-1) expression by qRT-PCR, which can be used to quantify melanoma metastases in murine spontaneous metastasis and autochthonous models. In addition to allowing metastatic burden to be evaluated, even where overt metastases are not present, our results also show that pulmonary metastases are not equally distributed amongst the lobes of the murine lung in individual animals, indicating that the quantification of pulmonary metastasis in murine models must take this heterogeneous distribution into account.

## 2. Materials and Methods

### 2.1. Cell Lines

The RheoSwitch inducible expression system [25] was introduced into B16-F10 cells. The resulting B16-RheoSwitch (B16-RS) cells were cultivated in DMEM containing 4.5 g/L glucose (Gibco/Thermo Fisher Scientific, Dreieich, Germany), 10% fetal bovine serum (Sigma Aldrich, Taufkirchen, Germany), and 1% penicillin/streptomycin (Gibco/Thermo Fisher Scientific). The cells were confirmed to be mycoplasma negative.

### 2.2. Ex Vivo Admixture Experiments

Murine lung tissue was admixed with 1 × 10^2^–1 × 10^6^ B16-RS cells transfected with RheoSwitch constructs, which enable the induced expression of the immediate early gene Ier2, and was dissociated mechanically in TRIzol, using metal beads with a TissueLyser II (QIAGEN, Hilden, Germany) for 5 min at 30 Hz, with the holder pre-cooled to −20 °C. In some experiments, individual lung lobes were admixed with 1 × 10^2^–1 × 10^5^ B16-RS cells. All samples were immediately processed for qRT-PCR analysis.

### 2.3. Quantitative Real Time PCR (qRT-PCR)

Total RNA was isolated using TRIzol reagent (Thermo Fisher Scientific), following the manufacturer’s instructions. RNA (1–2 μg) was treated with RNase-free DNase I (Thermo Fisher), followed by EDTA deactivation for 10 min at 65 °C. First strand cDNA was synthesized with random hexamer primers, using dNTP mix and RevertAid H Minus Reverse transcriptase (Thermo Fisher Scientific, Dreieich, Germany). qRT-PCR was performed in a Stratagene Mx3500P qPCR machine (Agilent, Waldbronn, Germany), using SYBR Select Master Mix, containing SYBR Green dye (Applied Biosystems/Thermo Fisher Scientific, Dreieich, Germany) or GoTaq qPCR Master Mix (Promega, Walldorf, Germany). The relative quantity of cDNA was estimated using the ΔΔCT method, and data were normalized to ribosomal protein 60S acidic ribosomal protein P0 (RPLP0). For the experiments in which Trp-1 levels in spontaneous metastases were quantified (Figures 2C and 3C), the data were additionally normalized to the mean Trp-1 signal in lungs of non-tumor-bearing mice, which was set at 1. The PCR program parameters were: 1 cycle of initial denaturation (95 °C, 2 min), followed by 40 cycles consisting of 95 °C for 15 s and 60 °C for 1 min, and a final cycle consisting of 95 °C for 1 min, 55 °C for 30 s, and 95 °C for 30 s.

The following forward and reverse primers, purchased from metabion (Steinkirchen, Germany), were used for qRT-PCR: RPLP0: 5′-GGA CCC GAG AAG ACC TCC TT-3′, 5′-GCA CAT CAC TCA GAA TTT CAA TGG-3′; Trp-1: 5′-GCT GGA GAG AGA CAT GCA GGA-3′, 5′-AGT GCA GAC ATC GCA GAC GTT-3′. Trp-2: 5′- TTA CGC CGT TGA TCT GTC AGA G-3′, 5′-TTG CGA AGC CTT CTG TAT TGA A-3′; RheoActivator element (RA): 5′-ACG CGC TAG ACG ATT TCG AT-3′, 5′- TCA AAC CCC TCA CCT CTG GA-3′.

### 2.4. Animals

C57Bl/6J mice were obtained from Charles Rivers Laboratories (Sulzfeld, Germany) and C57Bl/6JOlaHsd (C57BL/6J) mice were purchased from Envigo (Horst, Netherlands). C57Bl/6J;Tg(*Grm1*)/*Cyld^−/−^* mice [26] were used with the permission of Prof. Suzie Chen, who originally established the Tg(*Grm1*) line [27].

Mice were kept in groups of 4 in type III Makrolon filtertop cages (Tecniplast, Hohenpeißenberg, Germany) containing SAFE fs14 bedding (J. Rettenmaier & Söhne, Rosenberg, Germany). Rat/mouse extruded food (ssniff, Soest, Germany) and sterilized water acidified with HCl (pH: 2.8–3.1) was provided ad libitum. The specific-pathogen-free area was kept at 20 °C and 30–60% humidity on a 7:00–20:00 light cycle. The health status of the animals in the facility was routinely assessed by a commercial veterinarian laboratory (mfd Diagnostics, Wendelsheim, Germany) with serological examinations every three months (epizootic diarrhea of infant mice, mouse hepatitis virus, murine norovirus, minute virus of mice, Theiler´s encephalomyelitis virus, and *Pasteurella pneumotropica*) or annually (*Clostridium piliforme*, Mousepox, lymphocytic choriomeningitis virus, mouse adenovirus type 1 and type 2, *Mycoplasma pulmonis*, pneumonia virus of mice, Reovirus type 3, and Sendai virus).

### 2.5. Spontaneous Metastasis Assay

C57Bl/6 mice (9 weeks old) were injected with 1 × 10^5^ OPN-deficient B16-RS cells transfected with RheoSwitch constructs that enable the induced expression of the immediate early gene Ier2. The cells were resuspended in 100 μL PBS and injected s.c. into the flank. The data presented in this study are part of an experiment where 50 mg/kg of pharmacologically inert diacylhydrazine RheoSwitch ligand (Exclusive Chemistry Ltd., Obninsk, Russia) was administered i.p. daily to induce the expression of Ier2, starting from day 5 following cell transplantation. Control mice received an equivalent volume of DMSO vehicle. Tumor size was measured regularly in three dimensions using a caliper. The animals were sacrificed when the tumor size reached 2 cm in one dimension or if they became moribund. Lungs were explanted post mortem, inspected for visible metastases, photographed, and then flash frozen in liquid nitrogen. The frozen lungs were kept at −80 °C until RNA isolation. For RNA isolation, the frozen lungs were transferred into TRIzol, and further processed as described above.

### 2.6. Autochthonous Metastasis Model

C57Bl/6J;Tg(*Grm1*)/*Cyld^−/−^* transgenic animals were monitored once a week for the development of melanocytic lesions and melanoma growth. Mice aged 1 year were euthanized, lungs were explanted post mortem, inspected for visible metastases, photographed, and flash frozen in liquid nitrogen. The frozen lungs were kept at −80 °C until RNA isolation. For RNA isolation, the frozen lungs were transferred into TRIzol, and further processed as described above. For qRT-PCR analysis, half of each lung lobe was pooled into one sample. C57BL/6J mice aged 6 months and 1 year were used as controls.

### 2.7. Hematoxylin-Eosin Staining

Paraffin sections on glass slides were deparaffinized with three 5 min changes of RotiHistol and a descending series of 100%-96%-80%-70% ethanol. The slides were immersed for 30 s in Mayer′s hemalum solution (Merck) and washed for 10 min in running tap water for bluing. Staining with a 0.5% alcoholic eosin-Y solution (Merck, Darmstadt, Germany) was followed by a short wash in tap water, dehydration in an ascending series of 70%-80%-96%-100%-100% ethanol, three 5 min changes of Roti-Histol, and embedding in Eukitt mounting medium (Merck).

### 2.8. Statistical Analysis

Results are represented as the mean +SEM. Graphs for qRT-PCR experiments represent data from a minimum of 3 biological replicates, with each biological replicate being executed in 2 technical replicates. For multiple group analysis, one-way ANOVA was used in Prism 7 (GraphPad Software, San Diego, CA, USA).

## 3. Results

### 3.1. Trp-1 and Trp-2 Transcript Levels Correlate with Melanoma Cell Numbers Admixed with Lung Tissue Ex Vivo

**Figure 1:**
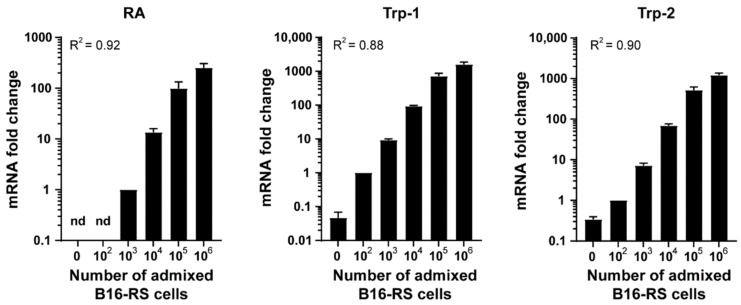
Trp-1 expression correlates with melanoma cell numbers admixed with lung tissue ex vivo. Metastatic load detection ex vivo. qRT-PCR analysis of RheoActivator (RA), Trp-1, and Trp-2 in murine lung tissue (1:1) admixed with 1 × 10^2^–1 × 10^6^ B16-RS cells. Data were normalized to RPLP0 and to the signal of 10^3^ cells (RS) or 10^2^ cells (Trp-1 and Trp-2) and represent mean + SEM, *n* = 3. R^2^: coefficient of determination. n.d.: not detected.

To allow easy and unambiguous quantification of melanoma metastatic burden, we determined the utility of Trp-1 and Trp-2 as melanoma cell markers, using the murine lung as a model organ. To this end, we first tested if the expression of Trp-1 and Trp-2 transcripts detected by qRT-PCR correlated with the number of B16-RS melanoma cells admixed with lung tissue. The presence of the RheoSwitch construct in the B16-RS cells enabled us to amplify and detect the RheoActivator element, which served as a tumor cell-specific marker, and was normalized to the expression of the 60S acidic ribosomal protein P0 (RPLP0), used as a house-keeping gene. We found that the level of RheoActivator signal detected by qPCR correlated with the number of admixed B16-RS cells in a linear manner, with a coefficient of determination of 0.92 (Figure 1). Similarly, Trp-1 and Trp-2 transcript levels also correlated with the number of admixed B16-RS cells, with comparable coefficients of determination for a linear correlation (Figure 1). The sensitivities of detection for both Trp-1 and Trp-2 were similarly high, since as few as 100 cells could be clearly detected by both markers (Figure 1). As the expression of Trp-1 was found to be higher than the expression of Trp-2, in further experiments, we focused on Trp-1 in order to optimize the detection of small numbers of tumor cells.

**Figure 2:**
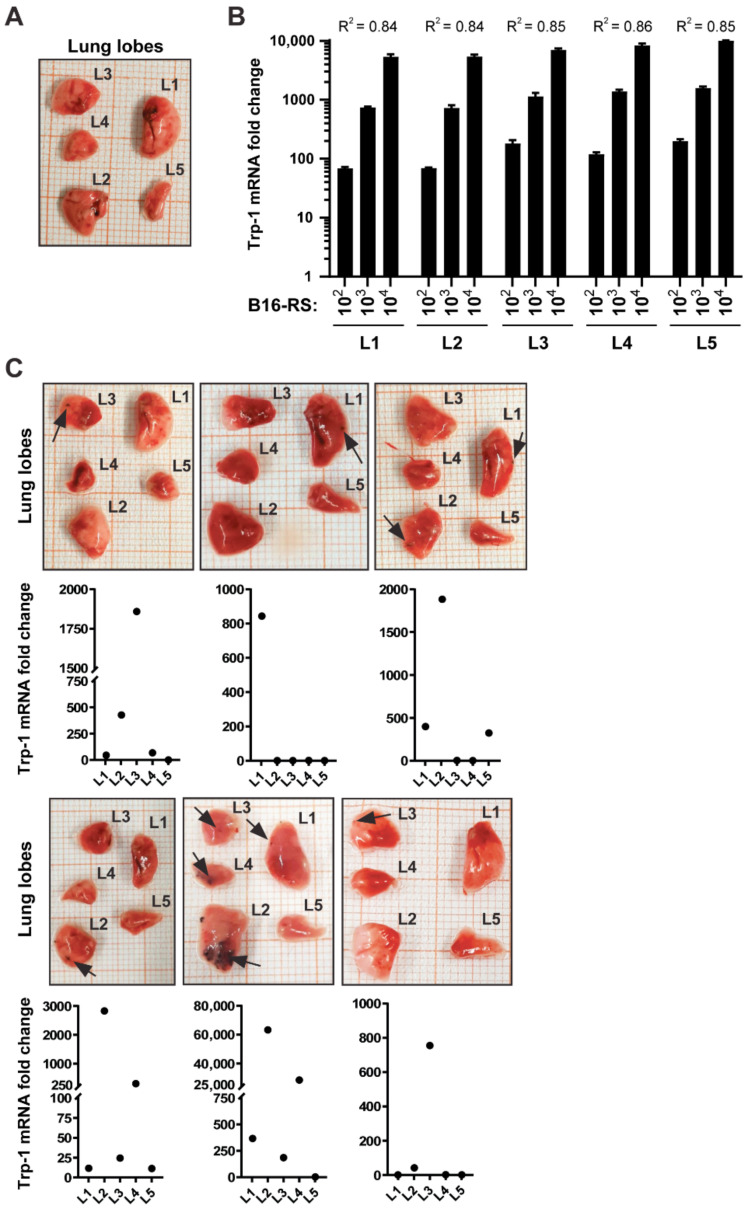
Trp-1 transcript levels correlated with metastatic burden in the lungs of experimental mice in a syngeneic mouse B16 model. (**A**) Representative image of a murine lung with isolated lung lobes: left lobe (L1), right caudal lobe (L2), right cranial lobe (L3), right middle lobe (L4), and accessory lobe (L5). Note that the arrangement of the lobes reflects the anatomical position, whereas the numbering corresponds to the size of the lobes, from largest to smallest. (**B**) qRT-PCR analysis of Trp-1 in samples containing 1 × 10^2^–1 × 10^4^ B16-RS cells admixed with individual lobes (L1–L5). Data were normalized to RPLP0 and to the mean Trp-1 signal in lobes without B16-RS cells. The graph shows mean Trp-1 expression + SEM, *n* = 3. R^2^: coefficient of determination. (**C**) B16-RS cells (1 × 10^5^) were injected subcutaneously into syngeneic mice (*n* = 6), and treated with either DMSO as a vehicle control (3 animals; top row) or RSL (3 animals; bottom row). The mice were sacrificed once the tumors reached a diameter of 2 cm in one dimension. Lungs were explanted and inspected for visible metastases. Trp-1 expression in the individual lobes was determined by qRT-PCR. Lung lobes with visible metastases (black arrows) are shown together with the corresponding Trp-1 expression in each of the five individual lung lobes (labelled L1 to L5, as in panel A), detected by qRT-PCR. Data were normalized to RPLP0 and to the mean Trp-1 signal in lobes of non-tumor-bearing mice.

Murine lungs consist of five individual lung lobes (right cranial lobe, right middle lobe, right caudal lobe, accessory lobe, and left lobe [28]) that differ in size (Figure 2A). To our knowledge, the incidence and distribution of metastases in each lobe has not been investigated so far. In order to determine whether Trp-1 expression might be useful to address this question for melanoma cells, we next assessed if the correlation between Trp-1 levels and melanoma cell number is maintained between the different lung lobes. To this end, different numbers of melanoma cells were admixed with individual lung lobes, and Trp-1 levels were then quantified and normalized to RPLP0. We observed a linear correlation with nearly identical coefficients of determination (0.84–0.86) for all lung lobes (Figure 2B). Together, these data show that Trp-1 transcription can be used as a quantifiable measure of the number of melanoma cells in all lung lobes.

### 3.2. B16-RS Lung Metastases Are Distributed Stochastically between the Lobes of the Mouse Lung

Next, we performed a spontaneous metastasis experiment with B16-RS cells in vivo, and investigated the utility of the qRT-PCR-based quantification of Trp-1 to determine metastatic burden in lung lobes where no visible overt metastases were present, as well as whether lung metastases were distributed equally amongst the lung lobes. To this end, B16-RS cells were subcutaneously injected into the flank of syngeneic mice. The animals were sacrificed once the primary tumors reached a diameter of 2 cm in one dimension. The lungs were then isolated, dissected, and the individual lobes were analyzed, first by eye for macroscopically visible metastases, and then via qRT-PCR for Trp-1 expression. We found that Trp-1 expression was highest in those lobes that showed overt macroscopically visible metastases (Figure 2C). Furthermore, a significant metastatic burden could be detected in specific lung lobes, even when no visible surface metastases were present (Figure 2C). Moreover, metastases were randomly distributed amongst the different lung lobes, and varied considerably from animal to animal, with some lobes being free of detectable tumor cells, while other lobes in the same animal had a high metastatic burden 3.3. Trp-1 Quantification Is a Sensitive Method for Detecting Pulmonary Metastases in Mice That Develop Autochthonous Melanoma.

**Figure 3:**
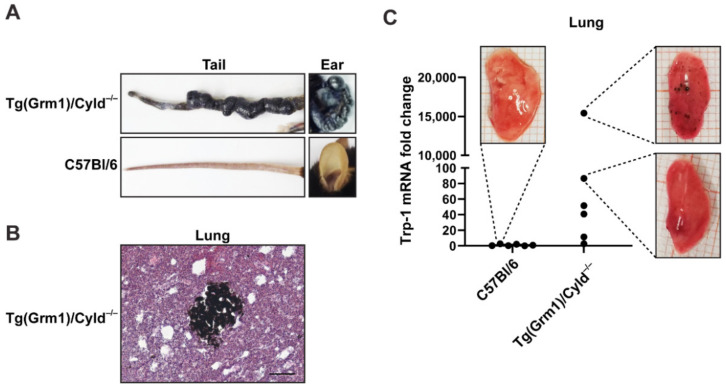
Quantitative detection of melanoma micrometastases in Tg(*Grm1*)/*Cyld^−/−^* transgenic mice. (**A**) Representative picture of melanomas detected on the tail and ear of 1-year-old C57Bl/6;Tg(*Grm1*)/*Cyld^−/−^* transgenic mice compared to C57Bl/6 control. (**B**) Hematoxylin/eosin (HE) staining of lung tissue from a C57Bl/6;Tg(*Grm1*)/*Cyld^−/−^* mouse containing a melanotic micrometastatic nodule. Scale bar, 500 µm. (**C**) qRT-PCR analysis of Trp-1 expression in lungs from C57Bl/6;Tg(*Grm1*)/*Cyld^−/−^* mice in comparison to C57Bl/6 controls. Data were normalized to RPLP0 and to the mean Trp1-1 signal in the C57Bl/6 controls; *n* = 6. The images show lung lobe L1 for the indicated data points.

Tg(*Grm1*)/*Cyld^−/−^* mice autochthonously developed melanoma mainly on the tail and ears (Figure 3A), due to an increased expression of the metabotropic glutamate receptor 1 (GRM1) under the control of the melanocyte specific Dct (Trp-2) promoter, and a lack of the tumor suppressor cylindromatosis (CYLD) [26]. The mice developed metastases in lymph nodes [26] and lung (Figure 3B,C). To assess the sensitivity of Trp-1 as a marker for quantifying pulmonary metastases in this context, we isolated lungs of Tg(*Grm1*)/*Cyld^−/−^* mice, documented macroscopically visible metastases, and tested their correlation with the expression levels of Trp-1 transcripts. Trp-1 expression in the lungs of C57Bl/6 wild type mice served as a baseline control. High Trp-1 expression in the lungs of Tg(*Grm1*)/*Cyld^−/−^* mice corresponded with the occurrence of visible metastases (Figure 3C). Lower levels of Trp-1 were also detected in lung lobes in which no visible metastases were present (Figure 3C), indicating that our protocol is sufficiently sensitive to detect disseminated tumor cells and micrometastases before they become macroscopically visible.

## 4. Discussion

Here, we described a protocol for the detection and quantification of melanoma metastases in the murine lung, based on quantification of Trp-1, a glycoprotein involved in melanin synthesis that is expressed in melanoma cells. This method allowed for the detection and quantification of low metastatic burden in both transplantable and autochthonous models of melanoma pulmonary metastasis, even in the absence of overt superficial metastases. Furthermore, our data showed that pulmonary metastases were stochastically distributed amongst the different lobes of the murine lung, an observation that has important ramifications for the accurate quantification of metastatic burden in experimental animal models.

A number of qRT-PCR-based approaches for the detection and quantification of metastases in rodent models have been described. After xenografting of human tumor cells into immunocompromised mice, quantification based on the amplification of DNA sequences that are specific to humans has been employed [11,12,14]. In systems where tumor cells have been genetically modified, qRT-PCR-based quantification of the transgene can be used for the evaluation of metastatic burden, as exemplified by transfected genes that encode for neomycin resistance [13], mCherry [17], and luciferase [18]. Comparatively few endogenous tumor-cell markers offer sufficient specificity and sensitivity for qRT-PCR-based quantification. Examples include GP-100 [15], Trp-1 [17], Trp-2 [15,17], Grm1 [29], and Her-2 [16]. These qRT--based protocols have been validated after either intravenous injection of tumor cells [14,15,18], or after orthotopic or subcutaneous implantation of tumor cells [11,12,13,14,16,17,18]. Here, we show that qRT-PCR-based analysis of Trp-1 transcription can also be used to quantify low pulmonary metastatic burden in the Tg(*Grm1*)/*Cyld^−/−^* autochthonous melanoma model, extending the range of metastasis models for which qRT-PCR-based strategies can be used.

Compared to other methods for the quantification of pulmonary melanoma metastases, the qRT-PCR-based approaches we described here offer a number of important advantages. The method facilitates a rapid, sensitive and accurate assessment of metastatic burden that is not possible with conventional visual inspection or histology-based methods. Furthermore, much lower levels of metastatic burden can be detected, compared to photonic imaging methods, as qRT-PCR is up to 10 times more sensitive than bioluminescence imaging [18,30]. Moreover, primary tumors often grow rapidly in spontaneous metastasis assays, which can require the termination of experiments before metastases have had time to develop fully, and can severely limit the utility of specific animal models for studying the process of metastasis. The sensitivity of the qRT-PCR-based quantification of Trp-1 means that metastatic burden can still be assessed at early stages of metastasis formation, extending the utility of these animal models. In addition, the sensitivity of the qRT-PCR method means that metastatic burden can be quantified when primary tumors are relatively small, reducing animal suffering. Similarly, the utility of Trp-1 quantification for the sensitive detection of metastasis in autochthonous melanoma models that we reported here means that metastasis formation can be assessed at an earlier stage, accelerating the time to endpoint analysis, thereby increasing experimental throughput. The method also facilitates assessment of metastasis formation in models in which rapid primary tumor growth necessitates termination of the experiment before overt visible metastases can be detected.

Although quantification of Trp-1 expression offers important advantages compared to other methods for quantifying metastatic burden, there are also a number of limitations. In contrast to live imaging techniques, qRT-PCR-based approaches allow only end-point analysis and no longitudinal assessment of metastasis in the same animal over time. Compared to quantification of superficial metastases or histology-based techniques, spatial information is lost, and it is not possible to determine whether a qRT-PCR signal is derived from a few large metastases or multiple small ones. Trp-1 expression may also be reduced in amelanotic melanoma metastases. Although amelanotic lesions can retain Trp-1 and Trp-2 expression [22,31], we have observed only very low Trp-1 expression in amelanotic lesions derived from YUMM melanoma cells ([32]). It has been shown that the expression of Trp-1 can be downregulated in invasive melanoma cells and in some melanoma metastases [20,33], and similar findings have been reported for Trp-2 [34]. Thus, reduced Trp-1 (or Trp-2) expression may limit the sensitivity of detection that can be achieved with our approach in some contexts. Nevertheless, Trp-1 protein expression has been demonstrated in the majority of melanoma metastases [35] and higher Trp-1 mRNA levels in skin and lymph node metastases have been correlated with worse clinical outcome for melanoma patients [36]. Furthermore, it was shown that in many cases Trp-1 protein cannot be detected in melanoma metastases even when its mRNA is expressed [36], which has been attributed to inhibition of Trp-1 translation by miR-155 [37]. Thus, the use of Trp-1 mRNA as a marker of disseminated melanoma cells could be possible even in metastases that are amelanotic or negative for Trp-1 by immunostaining.

Although we have focused on pulmonary metastasis in this study, the PCR-based quantification of Trp-1 can potentially also be used to quantify metastatic burden in other organs. Endogenous expression of Trp-1 in some organs may, however, limit the efficacy or sensitivity of such an approach. For example, Trp-1 and Trp-2 are expressed in the brain, but at much lower levels than those found in melanoma cells [38,39]. In addition, expression of Trp-1 in non-cutaneous melanocytes can generate some background signal in organs colonized by them, including the lung [40]. However, the endogenous Trp-1 levels in these organs are typically also much lower than those observed in melanoma cells, as demonstrated by our own results (Figure 2B,C and Figure 3C) and public datasets [40]). On the other hand, the approach we describe is likely to have limited utility for the detection of cutaneous dissemination of melanoma, due to the high numbers of Trp-1-expresing melanocytes in the skin.

Here, we report a dramatic animal-to-animal variation in the distribution of metastatic burden amongst the different lobes in the murine lung. This will have a pronounced impact on the reliability and accuracy of methods that assess pulmonary metastasis formation based on tissue sampling procedures, such as those that analyze metastasis formation in only a single lung lobe. The stochastic distribution of metastatic burden amongst the different lung lobes we observed strongly increases the risk that inappropriately high or low values for metastatic burden will be obtained for a given animal using these sampling methods, particularly in experimental settings in which few metastatic lesions develop. In turn, this will significantly increase variance in the experimental data, requiring the use of larger numbers of animals if statistically significant differences between experimental groups are to be obtained. Furthermore, non-concordant data may be obtained when, for example, one lobe is used for histology and another lobe is used for RNA-based analysis. Thus, analysis of pulmonary metastasis needs to take into account the distribution of metastatic lesions amongst the lung lobes, for which the qRT-PCR-based quantification we present here offers significant advantages.

## Data Availability

The primary data presented in this study are available on request from the corresponding author(s).
